# Pathogenic bacterial taxa constitute a substantial portion of fecal microbiota in common migratory bats and birds in Europe

**DOI:** 10.1128/spectrum.01948-24

**Published:** 2025-02-04

**Authors:** Anbu Poosakkannu, Yanjie Xu, Kati M. Suominen, Melissa B. Meierhofer, Iben H. Sørensen, Jesper J. Madsen, Betty Plaquin, Matthieu Guillemain, Emmanuel Joyeux, Oskars Keišs, Thomas M. Lilley, Aleksi Lehikoinen, Arto T. Pulliainen

**Affiliations:** 1 Institute of Biomedicine, University of Turku 8058 , Turku, Finland; 2 The Finnish Museum of Natural History, University of Helsinki 3835 , Helsinki, Finland; 3 Danish Hunters’ Association, Rønde, Denmark; 4 Department of Ecoscience, Aarhus University, Aarhus, Denmark; 5 Natural History Museum of Denmark, University of Copenhagen, Copenhagen, Denmark; 6 Office Français de la Biodiversité, Arles, France; 7 Office Français de la Biodiversité, Ile d’Olonne, France; 8 Institute of Biology, University of Latvia 61769 , Riga, Latvia; Michigan State University, East Lansing, Michigan, USA

**Keywords:** bat, wild bird, migration, microbiota, gut, dysbiosis, pathogen

## Abstract

Identifying the wildlife reservoirs of bacterial pathogens, spatially and temporally, is important for assessing the threats to human and the rest of the biosphere. Our objective was to study Europe-wide characteristics of the fecal microbiota of four highly mobile migratory vertebrates, that is, one bat (*Pipistrellus nathusii*) and three bird species (*Turdus merula*, *Anas platyrhynchos*, *Columba palumbus*). The 351 sample PacBio data set of almost the entire 16S rRNA gene with 438,997 amplicon sequence variants (ASVs) assigned 3,277 bacterial species. A significant proportion of the ASVs were assigned to bacterial genera having species pathogenic to human or animals. These pathogen ASVs accounted for 45% of all the ASVs and statistically were more frequent at higher latitudes and in younger age groups. In 36 samples, more than >90% of all the PacBio reads were assigned to these pathogenic genera. We designate to individuals of these samples a new term, that is, a pathogen bloomer. The pathogen bloomers, which did not display apparent macroscopic disease symptoms, were detected in Nathusius bat (*n* = 8; Finland and Latvia), blackbird (*n* = 6; Finland, Latvia and Denmark), and wood pigeon (*n* = 22; Finland and France), but not in mallard. Key species-level taxonomic assignments in the pathogen bloomers were the two well-known enteropathogens (*Campylobacter jejuni* or *Escherichia coli*) and one emerging enteropathogen (*Escherichia marmotae*). Our data imply that the studied common migratory vertebrates may contribute to the transmission of bacterial pathogens across the European continent.

**IMPORTANCE:**

The understanding of gut microbiota composition and dynamics in wild vertebrate populations, especially in highly mobile vertebrates, birds and bats, remains limited. Our study sheds light on the critical knowledge gap in how common pathogenic bacterial taxa of fecal microbiota are in migratory bats and birds in Europe. We found out that bacterial genera having species pathogenic to human or animals constituted a substantial portion of the fecal microbiota in all the studied host taxa. Most importantly, we identified asymptomatic individuals that were dysbiotic with bacterial pathogen overgrowth. These previously unknown pathogen bloomers appear as potent Europe-wide transmitters of bacterial pathogens, which cause, for example, diarrhea and bacteremia in human. Our findings may contribute to better understanding of seasonal disease hotspots and pathogen spillover risks related to migratory vertebrates.

## INTRODUCTION

The digestive tract of vertebrates hosts a large community of microbes, that is, the microbiota (bacteria, archaea, fungi, and viruses). Microbes in the gut are functionally involved in a number of key physiological processes, as exemplified by the broad range of laboratory animal- and human-based studies (1–3). Yet, the understanding of gut microbiota in wild vertebrate populations, its maintenance and variability, and its impact on the physiology, especially in highly mobile vertebrates, birds and bats, remains limited. This knowledge gap is also noteworthy given the avid interactions between these animals, which can annually travel thousands of kilometers, and the rest of the biosphere. Birds and bats may contribute to the inter-species and large-scale transmission of pathogens (4–8) with implications for wildlife conservation, agriculture, and human health.

A significant proportion of bird and bat species are migratory, facilitating the seasonal connection of individuals between diverse geographical habitats and populations. Bird- or bat-associated pathogens can therefore quickly spread over long distances (4, 8). Furthermore, with the ongoing climate change, resulting in shifts in population distributions (9, 10), migratory hosts might accelerate the introduction of pathogens into new regions (11). This emphasizes the importance of understanding the microbiota of migratory birds and bats, and the prevalence of pathogens within the microbiota across geographical locations and habitats. These kind of data could potentially increase the understanding of disease outbreaks and allow their prediction via modeling approaches (12).

Our knowledge of the composition of gut microbiota in bats and wild birds is still in its infancy (7, 13–15). To reduce this knowledge gap, this study focuses on examining the fecal bacteria of three common migratory bird species (Eurasian blackbird, hereafter blackbird, *Turdus merula*; wood pigeon, *Columba palumbus*, hereafter pigeon; mallard, *Anas platyrhynchos*) and one migratory bat species (Nathusius’ pipistrelle, *Pipistrellus nathusii*) across Europe. All species regularly interact with humans. All three bird species occur in urban areas, and pigeon and mallard, in particular, are popular game species. The Nathusius’ pipistrelle almost exclusively uses anthropogenic structures as roost sites. To achieve deep taxonomic resolution, we employed the PacBio long-read sequencing technique (16). This technique introduces distinct benefits, primarily attributed to its capability to capture nearly complete 16S rRNA sequences. This is a notable divergence from the shorter read lengths associated with other sequencing techniques, e.g., on the V4 region of 16S rRNA gene. To the best of our knowledge, only two previous microbiota studies on bats, that is, PacBio study in China (14) and Oxford Nanopore study in Spain (15), and none on wild birds have used the long-read sequencing techniques. In addition to the PacBio approach, we used traditional gene-targeted PCR with Sanger sequencing to elaborate some of the bacterial species-specific data. Our study provides insights into the fecal microbiota composition of four common migratory vertebrates with wide distribution ranges across the European continent.

## MATERIALS AND METHODS

### Sampling and research ethics

A team of scientists and hunters collected cloacal swab samples from three species of Eurasian migratory birds, namely the blackbird (*n* = 165), mallard (*n* = 135), and pigeon (*n* = 118) (File S1_1). The sampling was carried out across different geographical regions, including northern and eastern Europe (Finland, Denmark, and Latvia) and western Europe (France), covering various climate zones. The samples were collected either by capturing the birds passively using mist nets and other traps, e.g., funnel trap (in Pape, Latvia), or by shooting them during the hunting season. The age (young vs adult) and gender, except for pigeon samples, were assigned. After capturing or shooting, a cloacal swab sample was taken from each bird using Copan FLOQSwaps (520CS01 with regular tip or 501CS01 with a mini tip, Copan Diagnostics, Inc.). The FLOQSwaps were inserted into the cloaca to a depth of approximately 1 cm and rotated several times. After collection, the FLOQSwaps tips were cut and placed into 2 mL screw cap tubes (72.694.006, Sarstedt AG & Co. KG) containing 1 mL of RNALater solution. The RNALater solution was prepared in-house by dissolving 700 g of ammonium sulfate into a 1 L aqueous solution (pH 5.2) of 10 mM EDTA and 25 mM sodium citrate. The RNALater solution was stored at room temperature until used. The collected samples were transported to the laboratory and stored at −20°C. The sampling of a single species of migratory bat, the Nathusius’ pipistrelle (*n* = 161), was performed by a team of scientists (File S1_1). The bat fecal pellets were collected from eastern and northern Europe (Latvia, Finland, and Poland). Bats were caught using mist nets, harp traps, funnel trap (in Pape, Latvia), or by hand, and then temporarily placed in cotton or paper bags for a maximum of 20 minutes. During this time, fecal pellets were collected either from the cotton bag or during examination of the bats. The age (juvenile, subadult, adult) and gender were assigned. The fecal pellets were placed into 2 mL screw cap tubes (72.694.006, Sarstedt AG & Co. KG) containing 1 mL of RNALater solution. The collected samples were transported to the laboratory and stored at −20°C. The distribution ranges of the sampled taxa are described for blackbird at http://datazone.birdlife.org/species/factsheet/eurasian-blackbird-turdus-merula, for mallard at http://datazone.birdlife.org/species/factsheet/22680186, for pigeon at http://datazone.birdlife.org/species/factsheet/common-woodpigeon-columba-palumbus, and for Nathusius’ pipistrelle at https://www.iucnredlist.org/species/17316/22132621.

### DNA isolation

The bird cloacal swab samples in 1 mL of RNALater solution were vortexed to obtain a homogeneous suspension. In respect of bat fecal pellets, the RNALater solution was first discarded, and the fecal pellets underwent a wash with TE buffer (10 mM Tris, 1 mM EDTA, pH 7.4). Our approach with bat fecal pellets was based on pilot experiments where we consistently saw that homogenization of the bat fecal pellets directly in the RNALater storage solution resulted in poorer DNA yields as compared to the described method. Next, the bat fecal pellets were homogenized in 400 µL of PBS (phosphate-buffered saline, pH 7.4) using a Tissue Lyser for 3 minutes (first 90 seconds at 30,000 kHz, then tube holder turn with 180°, and finally another 90 seconds at 30,000 kHz). Subsequently, 500 µL of the bird and bat sample suspensions was used for DNA isolation with Macherey-Nagel NucleoSpin DNA Stool kit (740472.250) following the instructions of the manufacturer. Shortly, 500 µL of ST1 extraction buffer and 50 µL of elution buffer were used. The DNA concentrations were determined using the Invitrogen Qubit 4 Fluorometer with WiFi (Q33238) and Invitrogen Qubit dsDNA HS (High Sensitivity) kit (Q32851) according to the instructions of the manufacturer. The DNA samples were stored at −20°C.

### PacBio sequencing

A total of 263 bird samples, 88 bat samples, three DNA extraction controls, and one PCR water control were used for amplicon sequencing. The 16S rRNA gene amplicons were sequenced at University of Illinois, Roy J. Carver Biotechnology Center, DNA Services Laboratory. The universal primers 27F (AGRGTTYGATYMTGGCTCAG) and 1492R (RGYTACCTTGTTACGACTT) were used to amplify 16S rRNA gene fragments, utilizing the 2× Roche KAPA HiFi HotStart Ready Mix for high-fidelity PCR. Amplicons were converted to a library with the SMRTBell Express Template Prep kit 2.0. The pooled library was sequenced on 3 SMRTcell 8M on a PacBio Sequel IIe using the circular consensus long-read sequencing (CCS) mode and a 12-hour movie time.

### Bioinformatic analysis

The Roy J. Carver Biotechnology Center DNA Services Laboratory performed two rounds of demultiplexing using SMRTLink (v.11.0). The first round used the minimum passes (parameter set to –min-passes 3) to detect adapter sequences and minimum accuracy (parameter set to –min-rq 0.999) to output high-quality reads. The second round required at least two barcodes to be read and removed pairs with the same barcode (parameter set to –preset HIFI-ASYMMETRIC). The demultiplexed sequence data set was processed in-house using mothur software (v.1.48.0) as described by reference (17) with modifications for PacBio sequence analysis (File S1). The fastq.info command with the PacBio parameter set to true was used to convert the fastq files to fasta files. The make.group command was used to assign each sequence to its sample, followed by merging fasta files using merge.files command. The screen.seqs command was used to remove ambiguity (parameter set to 0) and homopolymer (parameter set to eight) sequences. The sequences were aligned using the Silva reference database (v.138.1) (18) to obtain sequences that overlap the same alignment coordinates (parameters set to start = 1,044 and end = 43,116) by filtering out overhangs at both ends. The pre.cluster command was used to group sequences up to two base pair differences, which were likely due to sequencing errors. The chimera.vsearch command was used to remove potential chimera sequences, with the dereplicate parameter set to true for the chimera search to remove any sequence flagged as chimeric in one sample from all other samples. Out of four controls (three DNA extraction and one 16S rRNA gene PCR water controls), two DNA extraction controls generated 7,906 reads with an average of 3,953 reads per control (File S1_3). The bioinformatics analysis of the two DNA extraction controls resulted in 980 ASVs (File S1_4), representing 7,808 reads (average of 3,904 reads per control). A total of 924 out of 980 ASVs were found specifically in the two DNA extraction controls, not in the bird and bat samples. Based on the decontam package analysis, any ASVs that were relatively more abundant in DNA extraction controls, some even up to 0.5% of the total reads, were considered potential contaminants and were removed from all samples. As a result, the remaining 56 (61,053 reads in bird and bat samples) out of 980 ASVs present in negative controls were removed from all samples (File S1_5). The eukaryotic chloroplast and mitochondria contaminants accounting for 4,726 ASVs (File S1_6) and 31,820 reads were also removed. The microeco package (v.0.14.0) was used to eliminate eukaryotic contaminants, including mitochondria and chloroplasts, while the decontam package (v.1.12.0) (19) with a prevalence approach threshold of 0.5% was used to identify and remove possible bacterial contaminants. The make.shared command was used to obtain the abundance data for the ASVs. The truncated SILVA database (v.138.1) was used as the source of subject sequences, and the query sequences were ASVs in the BLASTN 2.13.0+-based taxonomic assignments. The default parameters (blastn matrix 1–2, Gap Penalties: Existence: 0, Extension: 2.5) were used for the blast analyses, and the representative ASV sequences were assigned the taxonomy of the top hit. A phylogenetic tree was generated using qiime2 (20).

### Statistical analysis

The R software (v.4.2.1) (21) was used for the data analysis. The data set object was generated using the phyloseq (v.1.40.0) (22) and microeco (v.0.14.0) (23) packages, while the file2meco (v.0.3.0) package was used for object type conversion as needed. Taxonomic resolution analysis was performed using the microbiomeutilities package (v.1.00.17), and core microbiome analysis was conducted using the microbiome package (v.1.18.0). Rarefaction analysis was carried out using the mecodev package (v.0.2.0) (23), while Good’s coverage analysis was performed with the metagMisc package (v.0.0.4). The microeco package (v.0.14.0) was used for the following analyses: alpha diversity measures (Chao1 and Shannon diversity indices), ordination analyses (principal coordinate analysis, PCoA, with Bray-Curtis dissimilarities), determination of the significance of ordination clusters using permutational analysis of variance (PERMANOVA) and beta-dispersion tests, and identification of the ecological relevance of microbial communities using the Functional Annotation of Prokaryotic Taxa (FAPROTAX v.1.2.6) (24). The effect of host taxa on microbial communities was assessed using the entire data set, and subsets of data from each host taxa were used to investigate the effects of other variables, such as country, host age, and gender. Similarly, core microbiome analysis and identification of ecological relevance of microbial communities were performed on subsets of data of each host taxa. In the context of statistical comparisons, the subsampling process was applied to ensure an equivalent sampling depth, that is, each sample used for statistical analysis contains an equal number of sequences. Specifically, 1,000 sequences were randomly chosen per sample, based on the validation (Fig. S2; File S1_9).

### Targeted PCR-based screening

Targeted PCR assays were implemented/developed, including a multiplex PCR that used genus-specific primers for *Campylobacter* (16S rRNA gene) and *Salmonella* (invA gene), species-specific primers for *Yersinia enterocolitica* (ail gene), and strain-specific primers for Shiga toxin-producing *Escherichia coli* (Stx2 type A gene). Additionally, a duplex PCR was developed that used genus-specific primers for *Bartonella* (ssrA gene) and *Borrelia* (OspA gene). We used chromosomal DNA of *Campylobacter jejuni* strain DSM4688 (GenBank CP019838), *Salmonella enterica* serovar Typhimurium strain SL1344 (GenBank FQ312003), *Yersinia enterocolitica* strain YeO3-R1 (no genome sequence available), *Bartonella quintana* strain JK31 (GenBank FQ312003), and *Borrelia garinii* strain SKB40 (no genome sequence available), as well as synthetic DNA fragment (Eurofins Genomics) of Stx2a Shiga toxin gene (GenBank accession code CP038428) as positive controls for the PCR. After detecting the presence of *Campylobacter* in the multiplex PCR, additional screening was conducted using genus-specific primers targeting the *rpoB* gene. Primers and the PCR protocol are described in File S5_44. A total of 418 cloacal swab samples from birds and 161 fecal pellet samples from bats were screened using the multiplex and duplex PCR assays. The positive samples were Sanger sequenced (Eurofins Genomics). The Sanger sequence trace files in AB1 format were initially examined through visual inspection using Geneious Prime software (version 2022.2.2). This inspection aimed to identify and eliminate low-quality bases, and, subsequently, high-quality forward and reverse sequences were extracted. Next, a pairwise alignment was performed using Geneious Prime software (version 2022.2.2) to generate consensus sequences for each sample. For the multiple sequence alignment step in taxonomy, we used the default parameters in Geneious Prime. Specifically, the alignment was conducted with the following settings: alignment type set to global alignment with free end gaps, a cost matrix of 65% (5.0/−4.0), a gap open penalty of 12, a gap extension penalty of 3, and refinement iterations were set to 2. Following the multiple sequence alignment, a phylogenetic tree was constructed using the Geneious tree builder tool. The Tamura-Nei genetic distance model and the UPGMA tree build method were employed for this purpose. Additionally, a bootstrap resampling method with 100 replicates and a 50% threshold level was applied to enhance the reliability of the consensus tree. The phylogenetic tree was visualized and annotated using ggtree (v.3.8.0) in R software.

## RESULTS

### A diverse array of mostly host taxa-specific amplicon sequence variants

**Fig 1:**
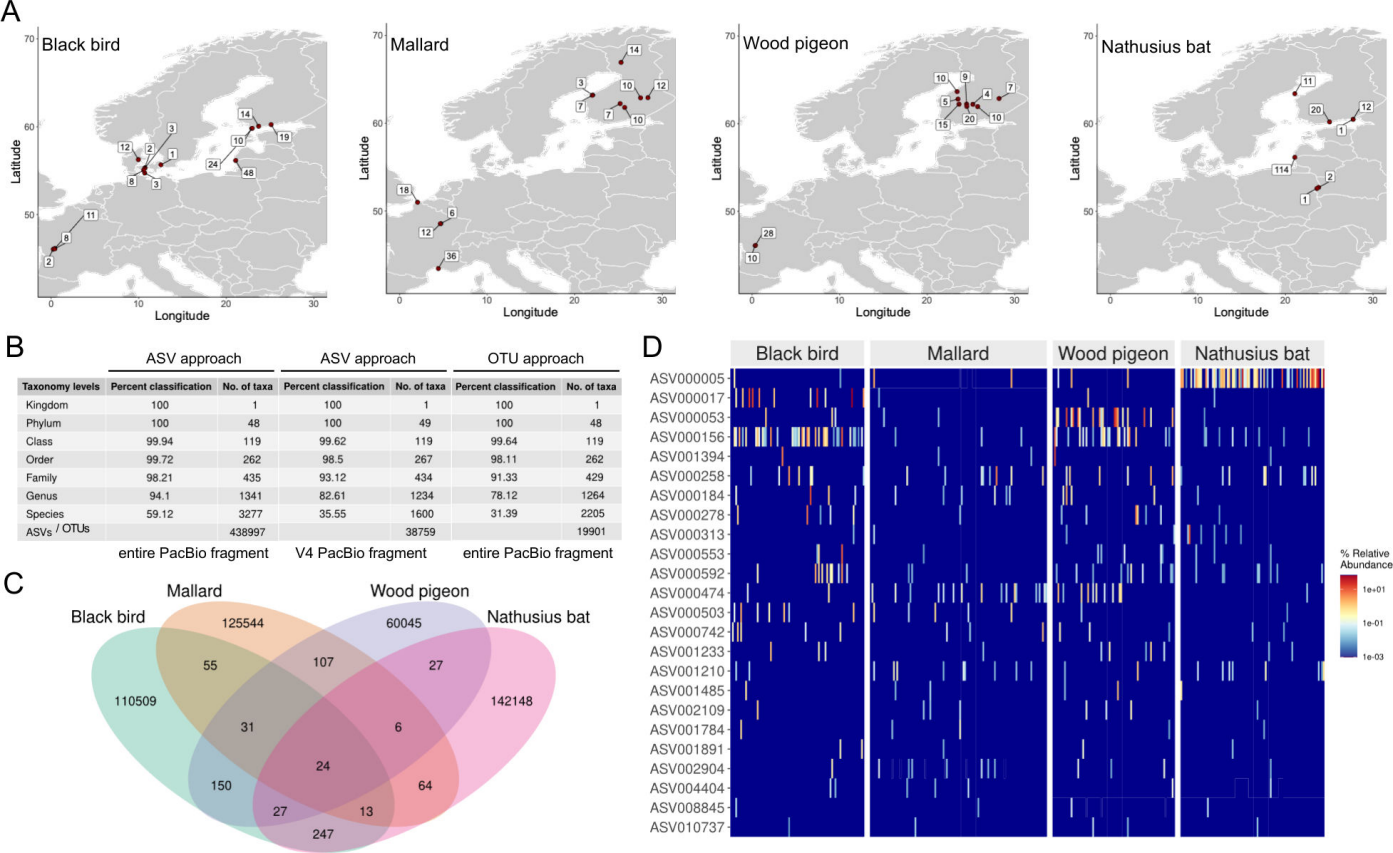
A diverse array of mostly host taxa-specific fecal amplicon sequence variants. (A) The sampling maps across Europe (locations, number of individuals). The maps were created with the R package ggmap. (B) The taxonomy resolution of the ASVs, e.g., 100% of the ASVs were assigned at the phylum level, representing 48 phyla. The analysis was done with the entire PacBio fragment (1,439 bp), and the shorter V4 region of the PacBio fragment (297 bp) that is typically employed in other sequencing platforms, e.g., referring to nucleotide positions 510 to 806 of *Escherichia coli* strain K-12 MG1655 (GenBank ON394478.1). The taxonomy resolution is also displayed at the OTU level with the entire PacBio fragment using the canonical 97% sequence homology clustering to obtain a single unique OTU. (C) The Venn diagram of unique and shared ASVs among the four host taxa. The integer is the number of shared ASVs. (D) Heat map of the percent relative abundancies (number of ASV-specific reads in a sample / all reads in a sample * 100) of the 24 ASVs detected in at least one individual of all the host taxa.

A total of 418 bird cloacal swab and 161 bat fecal pellet DNA samples, respectively, were isolated across Europe (Fig. 1A; File S1_1). A subset, that is, 263 bird and 88 bat samples, was subjected to PacBio sequencing. Out of these 351 samples, 349 generated a total of 3,124,276 reads (File S1_2). Two samples did not yield any raw reads, likely due to issues such as low DNA quality or unsuccessful library preparation. The bioinformatics-based quality control resulted in 444,371 unique reads, that is, ASVs, representing 2,765,308 reads. The final data set, after bioinformatics-based extraction of eukaryotic contaminant ASVs as well as ASVs present in the DNA extraction and 16S rRNA amplification controls (File S1_3–6), was composed of a total of 2,672,435 reads (average of 7,657 reads per sample) and 438,997 ASVs. The taxonomy resolution of these ASVs is shown in Fig. 1B, e.g., 3,277 species were identified. In comparison, only 1,600 species were identified based on the trimmed 297 bp V4 16S rRNA region. Also, much weaker taxonomic resolution was achieved with the full-length PacBio fragment using the operational taxonomic unit (OTU) approach, e.g., 2,205 species. Therefore, we used the full-length 16S rRNA ASV approach throughout the rest of the study. A total of 24 ASVs were detected in all the host taxa (Fig. 1C; File S1_7). Their percent relative abundance profile is shown in Fig. 1D. A total of 31 ASVs were detected in all the bird taxa (Fig. S1; File S1_8). Taken together, PacBio sequencing detected a diverse array of 16S rRNA ASVs with most of the ASVs showing host taxa specificity.

### Dominance of low relative abundance amplicon sequence variants

The downstream statistical analysis of the 438,997 ASVs involved subsampling. First, a rarefaction analysis was performed by plotting ASV richness in relation to observed total reads. The results showed that the sequencing depth was sufficient to cover most of the bacterial diversity (Fig. S2). Furthermore, the Good’s coverage, defined as 1 − (F1 / *N*), where F1 is the number of singleton ASVs and *N* is the sum of counts for all ASVs, demonstrated that all the samples except one had 97 to 100% of sequencing completeness (File S1_9). According to the rarefaction and Good’s coverage analyses, using subsampling for 1,000 sequences per sample was deemed sufficient to adequately represent most of the bacterial diversity. The rarefaction and Good’s coverage analyses excluded samples of nine pigeons and one mallard due to having fewer than 1,000 sequences. Upon subsampling, the ASV count was reduced from 438,997 to 70,001. Therefore, a large proportion of the ASVs were present at low relative abundance.

### Composition of fecal microbiota differs between the host taxa

**Fig 2:**
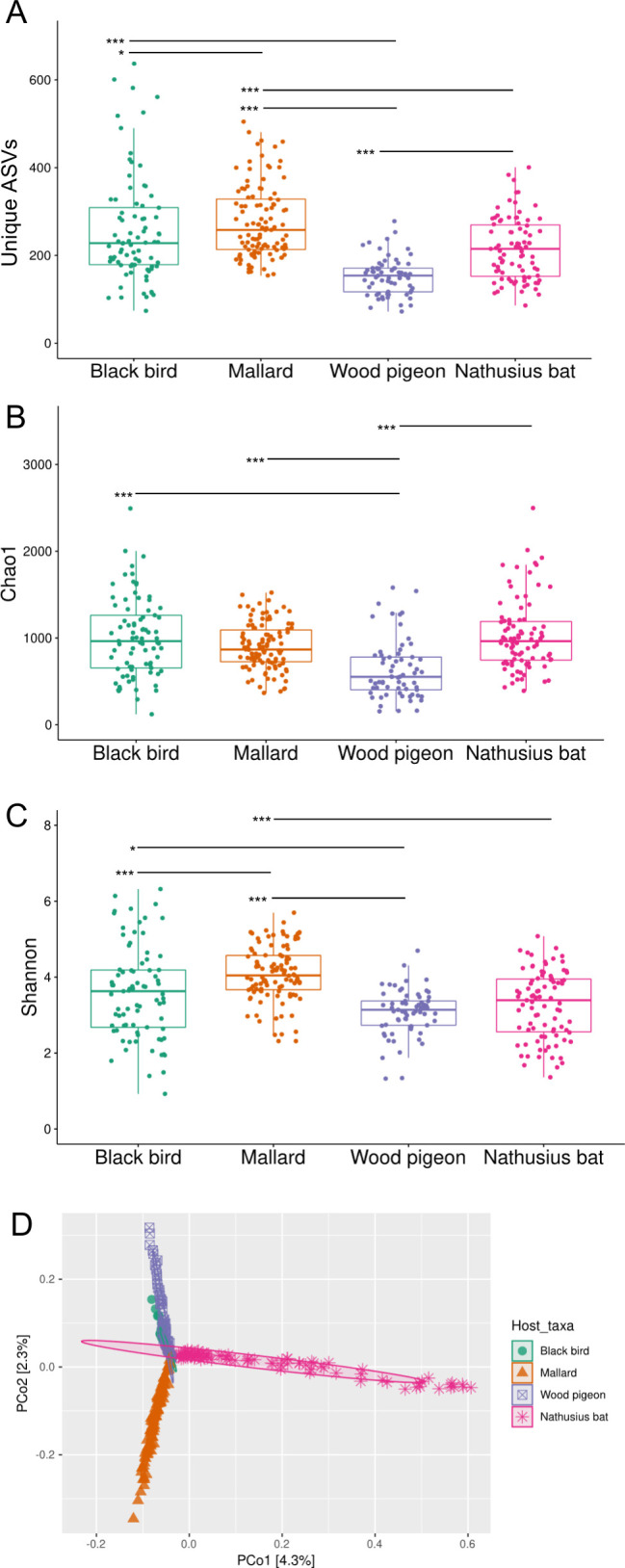
The composition of fecal microbiota differs between the host taxa. Three analyses are shown to compare host taxa differences in microbiota richness (A–C, alpha diversity metrics), and one analysis to compare host taxa differences in microbiota composition (D, beta diversity, see also File S1_10). The box plots of the number of unique ASVs (A) and Chao1 (B) as well as Shannon alpha diversity (C) indices at ASV level in different host taxa. Each point represents a single sample. A total of 339 subsampled samples were included in the analysis. The significance estimates were calculated by Wilcox rank sum test. Asterisks designate significance level (***≤ 0.001, **≤ 0.01, *≤ 0.05). (D) The PCoA ordination plot of the 339 subsampled samples. Each point represents a single sample. The ellipses are drawn based on the 95% confidence intervals.

First, we used three commonly used measures of alpha diversity, number of unique ASVs as well as the Chao1 and Shannon diversity indices, to investigate host taxa differences in microbiota richness (Fig. 2A through C). The number of unique ASVs and the Chao1 indices of pigeon were significantly lower as compared with any other host taxa (Fig. 2A and B). However, the diversity difference between pigeon and Nathusius’ pipistrelle was not observed when looking at Shannon indices (Fig. 2C). The number of unique ASVs and the Shannon indices of mallard were higher compared to any other host taxa (Fig. 2A and C). Overall, the alpha diversity data imply that the microbiota of different host taxa have richness differences, although the indices yielded somewhat contradictory results. Next, we conducted beta diversity analysis to investigate host taxa differences in microbiota composition. We used PCoA as well as PERMANOVA and beta dispersion analysis with Bray-Curtis dissimilarity metrics. We detected clear PCoA clustering pattern of mallard and Nathusius’ pipistrelle away from the other host taxa (Fig. 2D). Blackbird and pigeon overlapped to a large extent (Fig. 2D). The PERMANOVA confirmed the significance of the mallard and Nathusius’ pipistrelle PCoA clustering pattern, but it also separated blackbird and pigeon (File S1_10). We also conducted alpha, and, in particular, beta diversity analyses on the country, age, and gender groups (Fig. S3 to 5; File S1_11–12). Clear PCoA clustering patterns were not detected in any of the host taxa, indicating that country, age, and gender did not strongly influence the microbiota composition. Overall, the beta diversity analysis implied that the composition of fecal microbiota differed between the host taxa.

### Low within-host taxa conservation level of bacterial taxa

**Fig 3:**
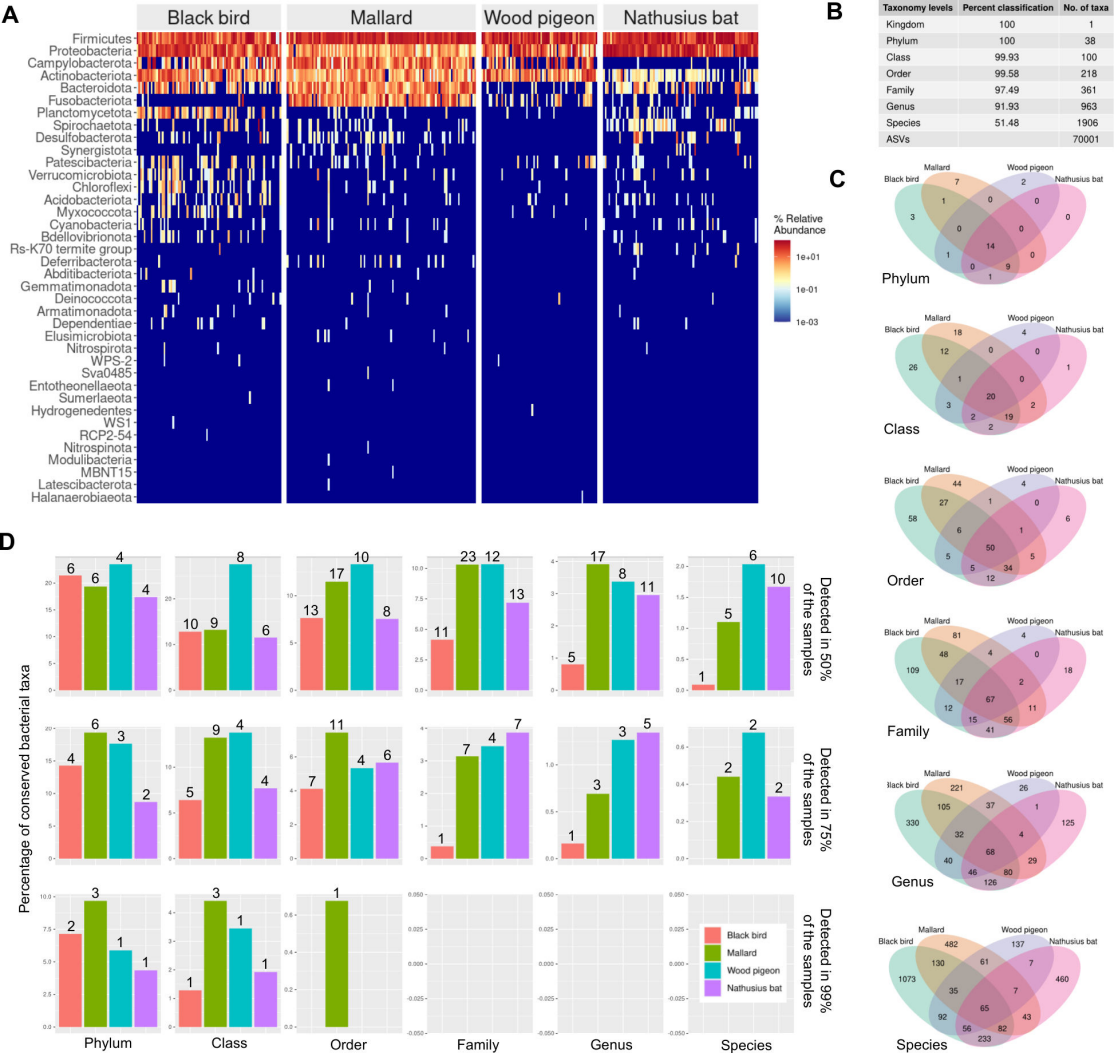
A limited number of fecal bacterial taxa conserved in studied individuals. The subsampled data set was used for all the analyses. (**A**) Heat map of the percent relative abundancies (number of ASV-specific reads in a sample / all reads in a sample * 100) of phyla identified in different host taxa. (**B**) The taxonomy resolution of the ASVs, e.g., 100% of the ASVs were identified at the phylum level, representing 38 phyla. (**C**) The Venn diagrams at different taxonomic resolutions of unique and shared taxonomic assignments. The integer is the number of shared taxonomic assignments. (**D**) Conservation of bacterial taxa at different taxonomic resolutions in different host taxa (File S1_13). The figure displays bacterial taxa that were present in 50%, 75%, or 99% of the samples. The *y*-axis percentages are calculated by dividing the number of conserved bacterial taxa of a given taxonomic resolution with the total number of bacterial taxa of the same taxonomic resolution in each host taxa * 100. The numbers above the bars refer to the frequency of conserved bacterial taxa, e.g., 75% of the mallard samples contained the same two bacterial species.

Based on the subsampled ASV data set, 38 bacterial phyla were assigned. The top 5 most common phyla were Firmicutes, Proteobacteria, Campylobacterota, Actinobacteriata, and Bacteroidota (Fig. 3A). Fourteen bacterial phyla were detected in all the host taxa (Fig. 3A through C). No bacterial taxon of any host taxa was present in all the individuals (Fig. 3D, File S1_13), not even at the phylum level. However, some relatively common taxa at high taxonomic resolution were identified. For example, two bacterial species were found in 75% of the samples from Nathusius’ pipistrelle (*Malacoplasma muris* and *Erwinia persicina*), mallard (*Campylobacter canadensis* and Bisgaard Taxon 44 of the *Pasteurella* family), and pigeon (*Enterococcus columbae* and *Escherichia coli*) (File S1_13; Fig. 3D). No bacterial species were so common in the blackbird samples. The low conservation level was characteristic for blackbird as compared to the other host taxa, also at lower taxonomic resolutions. Overall, the within-host taxa conservation analysis demonstrated that fecal samples had a limited number of common bacterial taxa, even at low taxonomic resolutions.

### Fecal microbiota have human and plant pathogenic potential

**Fig 4:**
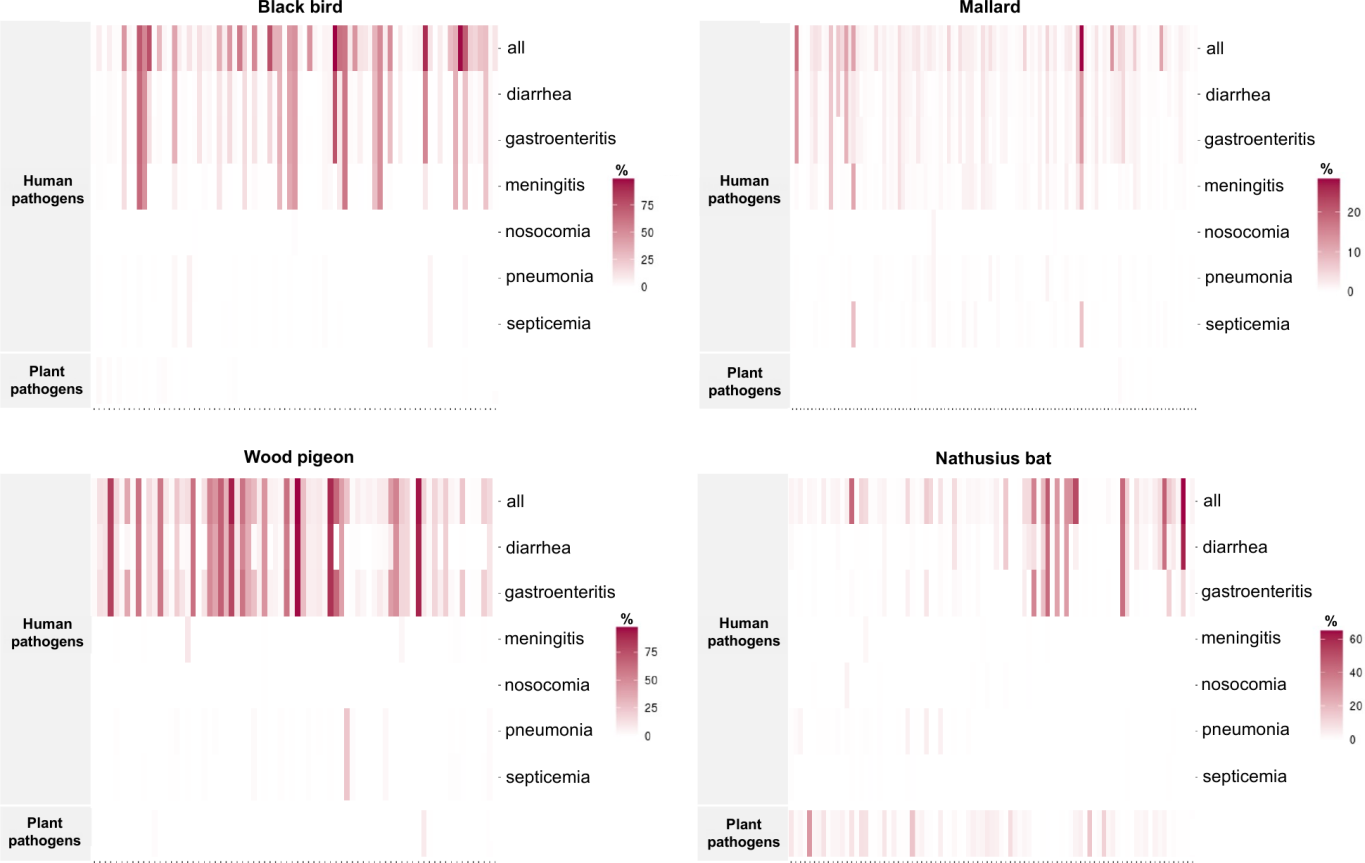
The fecal microbiota have human and plant pathogenic potential. The heat maps display the percentage of ASVs within each sample that were classified into different categories using the FAPROTAX database. The *x*-axis represents the individual samples, while the *y*-axis shows the FAPROTAX annotated categories.

To determine potential pathogenic associations, the subsampled ASV data set was mapped to the FAPROTAX database. Strong human pathogenic potential was detected (Fig. 4). Up to 80% of the unique ASVs per sample were assigned as human pathogens in blackbird and pigeon samples. With Nathusius’ pipistrelle and mallard, these maximum values were 60% and 40%, respectively. Blackbird had the highest number of unique ASVs assigned as potential human pathogens, with a count of 3,550. Pigeon followed with 1,994 ASVs, then Nathusius’ pipistrelle with 1,397 ASVs, and mallard with 641 ASVs (File S1_14–17). A higher percentage of ASVs were mapped to the intestinal infection-related pathogens in all host taxa as compared to meningitis-, pneumonia- or septicemia-related pathogens (Fig. 4). In addition, ASVs that specified plant pathogens, such as *Erwinia persicina* (417 unique ASVs), *Pantoea agglomerans* (102 unique ASVs), *Erwinia rhapontici* (15 unique ASVs), *Pseudomonas syringae* (five unique ASVs), and *Clavibacter michiganensis* (one unique ASVs) were detected (Fig. 4; File S1_18). It is noteworthy that 494 ASVs, out of the total 546 unique plant pathogen specifying ASVs, were detected in the Nathusius’ pipistrelle samples. Also, 75% of the Nathusius’ pipistrelle samples were positive for plant pathogen ASVs with 0.263 (*Erwinia persicina*) as the highest ASV relative abundance value. Taken together, the data imply that fecal bacteria of all host taxa have human pathogenic potential, and fecal bacteria of Nathusius’ pipistrelle, in particular, have plant pathogenic potential.

### Identification of pathogen bloomers

**Fig 5:**
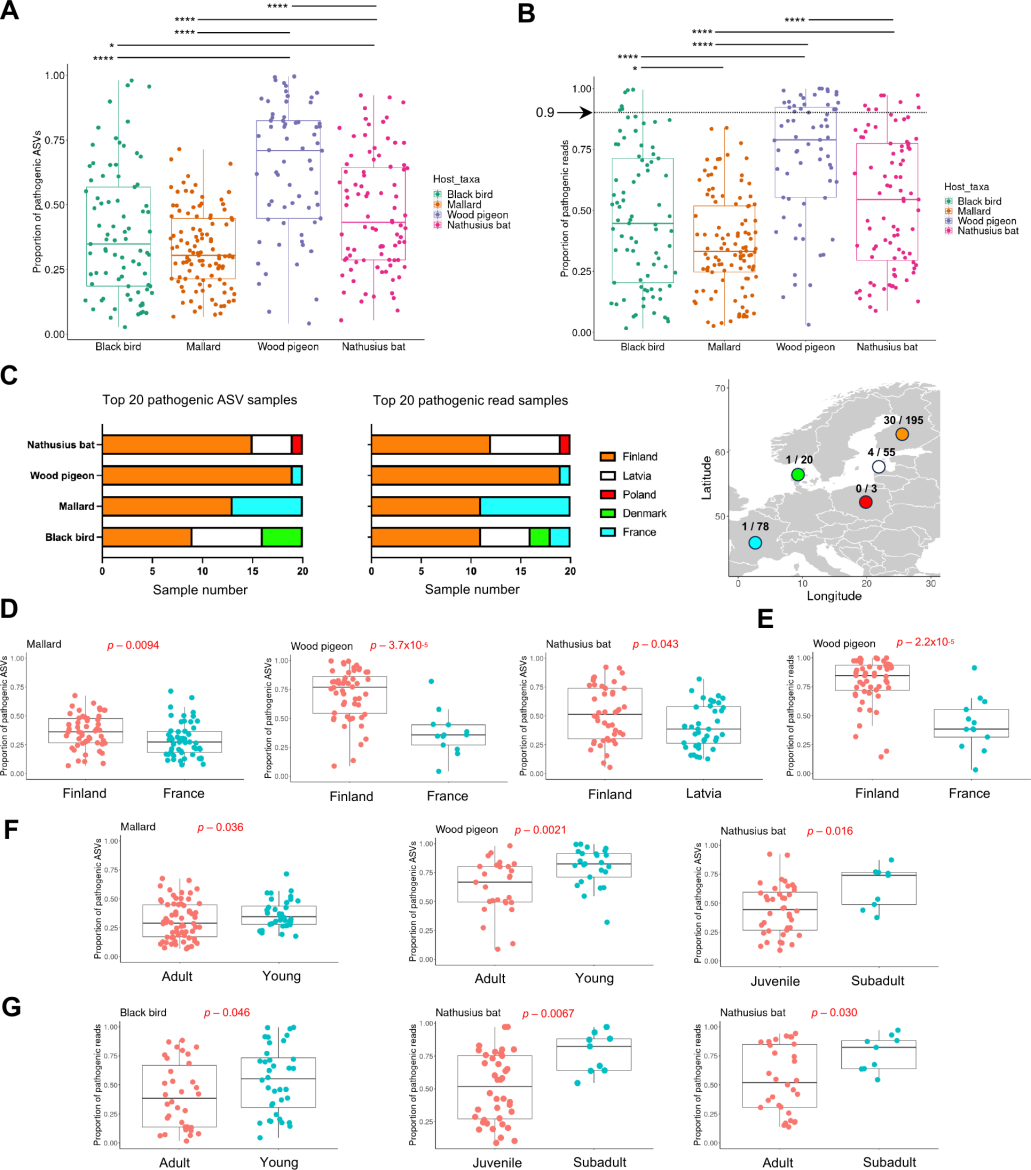
Identification of pathogen bloomers. The subsampled data set was used for all the analyses. (**A**) The proportions of pathogen genera ASVs (pASVs) in different host taxa (number of unique pASVs in a sample / number of all unique ASVs in the same sample). Each dot refers to one sample. Asterisks designate significance level (****≤ 0.00001***≤ 0.001, **≤ 0.01, *≤ 0.05). (**B**) The proportions of pathogenic reads in different host taxa (number of pathogen reads in a sample / number of all reads in a sample). Each dot refers to one sample. The arrow marks the position of a cut-off value set to score the pathogen bloomers. Asterisks designate significance level (****≤ 0.00001***≤ 0.001, **≤ 0.01, *≤ 0.05). (**C**) Geographic locations and distributions of the individuals with 20 of the highest proportions of pASVs and pathogen genera reads. Values in the map refer to the number of pathogen bloomer samples per country and the number of BacBio-sequenced samples per country. The map was created with the R package ggmap. (**D–G**) Statistical comparisons of the proportions of pASVs and pathogen genera reads in different countries (**D, E**) and age groups (**F, G**). Of all the executed comparisons (country, age, gender), the sub-figures D–G display differences that were statistically significant (*P*-value ≤ 0.05). All the executed comparisons are described in File S1_30–35. The significance estimates were calculated by Wilcox rank sum test.

To further analyze how abundant the pathogenic genera ASVs (hereafter pASVs) are, we conducted a pathogen-focused sub-analysis. We included 66 genera to our analysis, that is, genera, which by definition, include at least one species harmful to human or animals (File S1_19). We identified 199,531 pASVs, representing 45% of all the ASVs. The pASVs accounted for 1,417,269 reads, which is 53% of the total reads. To statistically compare the proportions of pASVs and pathogen genera reads, we used the subsampled data set. The pASV output reflects the pathogen richness (File S1_20–23), whereas the pathogen genera read output reflects the overall pathogen load (File S1_24–27). The pigeon had the highest proportion of pASVs (Fig. 5A; File S1_28), and also the highest proportion of pathogen genera reads (Fig. 5B; File S1_29). Almost all the detected reads in some pigeons were classified as pASVs (Fig. 5B). Here, we designate to these individuals a term pathogen bloomer, which refers to an individual where proportion of pathogen genera reads is >0.9. Altogether, 22 of such pigeons were detected. Similar trend was apparent with blackbird (*n* = 6) and Nathusius’ pipistrelle (*n* = 8), but not with mallard. Geographically, higher proportions of pASVs and pathogen genera reads were detected at higher latitudes (Fig. 5C), and statistically four differences between countries were detected (Fig. 5D and E; File S1_30–31). Also, there was a statistical trend toward higher pASV and pathogenic genera read proportions in the younger age groups (Fig. 5F and G; File S1_32–33). We did not detect gender differences (File S1_34–35). Taken together, a significant proportion of ASVs in all host taxa were assigned to pathogenic genera. Reads of 36 blackbird, pigeon, and Nathusius’ pipistrelle individuals, the so-called pathogen bloomers, were almost entirely classified to pathogenic genera.

### Pathogen bloomers are characteristically dysbiotic

**Fig 6:**
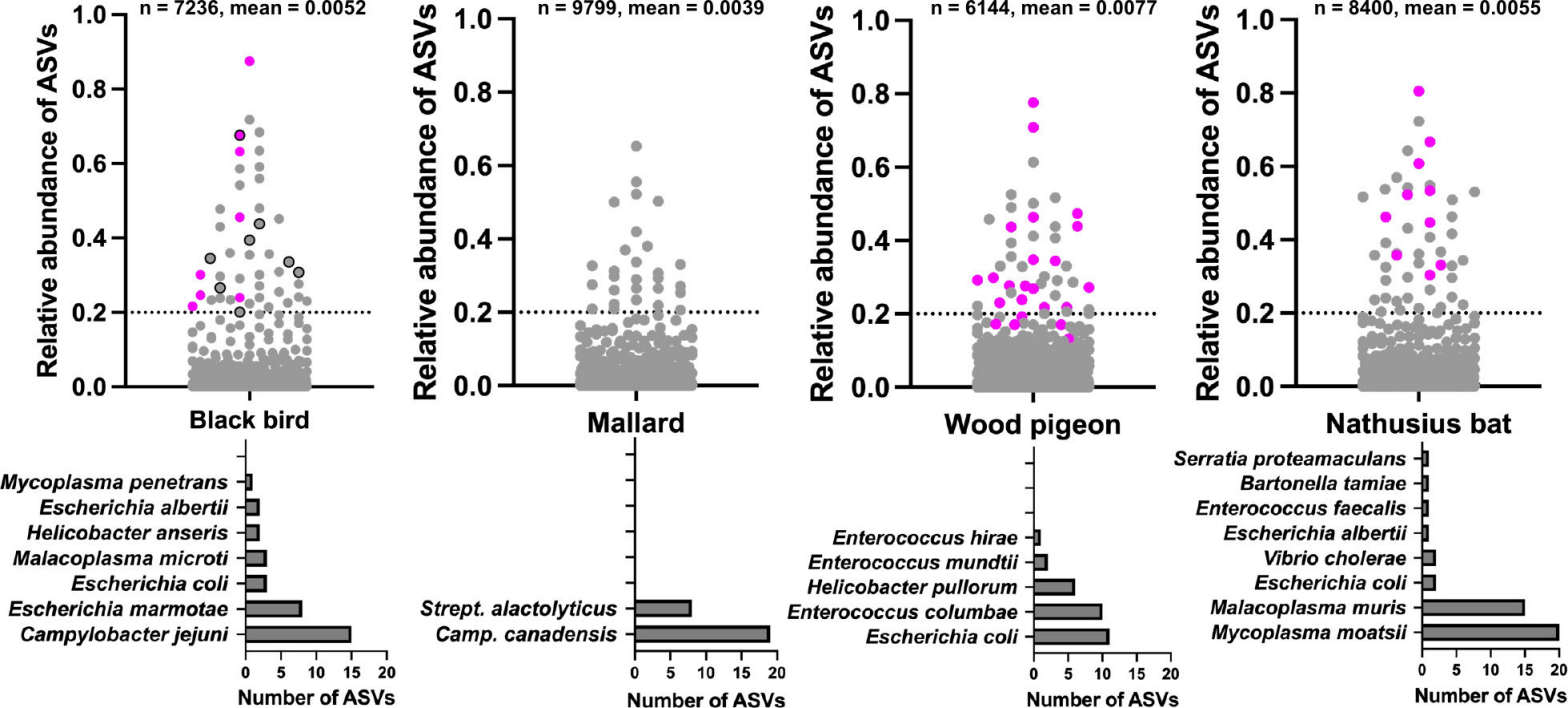
Pathogen bloomers are characteristically dysbiotic. Analysis of the relative abundancies (dot blots) and taxonomic designations (bar graphs) of pASVs. The subsampled data set was used for all the analyses. Relative abundancies of pASVs (number of pASV-specific reads in a sample / number of all reads in a sample) were calculated and visualized in the dot plots. Each dot represents one pASV. The same pASV appears multiple times if it has been detected in more than one sample. The total number of pASV detections is shown within each sub-panel with a mean relative abundancy (number of pASV-specific reads in a sample / all reads in a sample). The pink dots refer to the pASVs that were detected in the pathogen boomers, that is, individuals where proportion of pathogen genera reads was >0.9 (see Fig. 5B). The dots, which are circled in black, refer to ASVs that specify *Escherichia marmotae*, an example of an enteric human pathogen. The dashed line at 0.2 is a cut-off for a pASV, which is regarded in this study as having a high relative abundancy value. The bar charts show those bacterial species that were identified based on the pASVs located above the 0.2 cut-off dashed line. The number of ASVs refer to the total number of unique pASVs per given species.

Next, we analyzed if the high proportion of pathogenic reads in a sample is caused by one pASV with high relative abundance value or by multiple pASVs. The relative abundancies of pASVs were mostly low with an all-host-taxa mean of 0.005 (Fig. 6; File S2_36–39). However, the pathogen bloomer samples (36 in total), except from five pigeons, contained pASVs with relative abundancy values higher than 0.2 (Fig. 6). In respect of the five pigeon pathogen bloomer samples below the 0.2 cut-off, the pASV relative abundancies were still relatively high (0.192, 0.172, 0.170, 0.170, 0.131). Of note, we deliberately used a stringent 0.2 cut-off, that is, 20% of the reads in a sample had to be specific for a given pASV to be scored as high relative abundance pASV. Notable taxonomic species assignments with the high relative abundancy pathogen bloomer pASVs were the well-known enteric pathogens *Campylobacter jejuni* (0.632, blackbird) and *Escherichia coli* (0.474, pigeon) as well as the emerging enteric pathogen *Escherichia marmotae* (0.676, blackbird) (Fig. 6). Taken together, the high proportion of pathogenic reads in pathogen bloomer samples was associated with one pASV with high relative abundance value. The data imply that the pathogen bloomers are characteristically dysbiotic with single pathogen overgrowth.

### Dominance of host-specific pathogenic genera amplicon sequence variants

**Fig 7:**
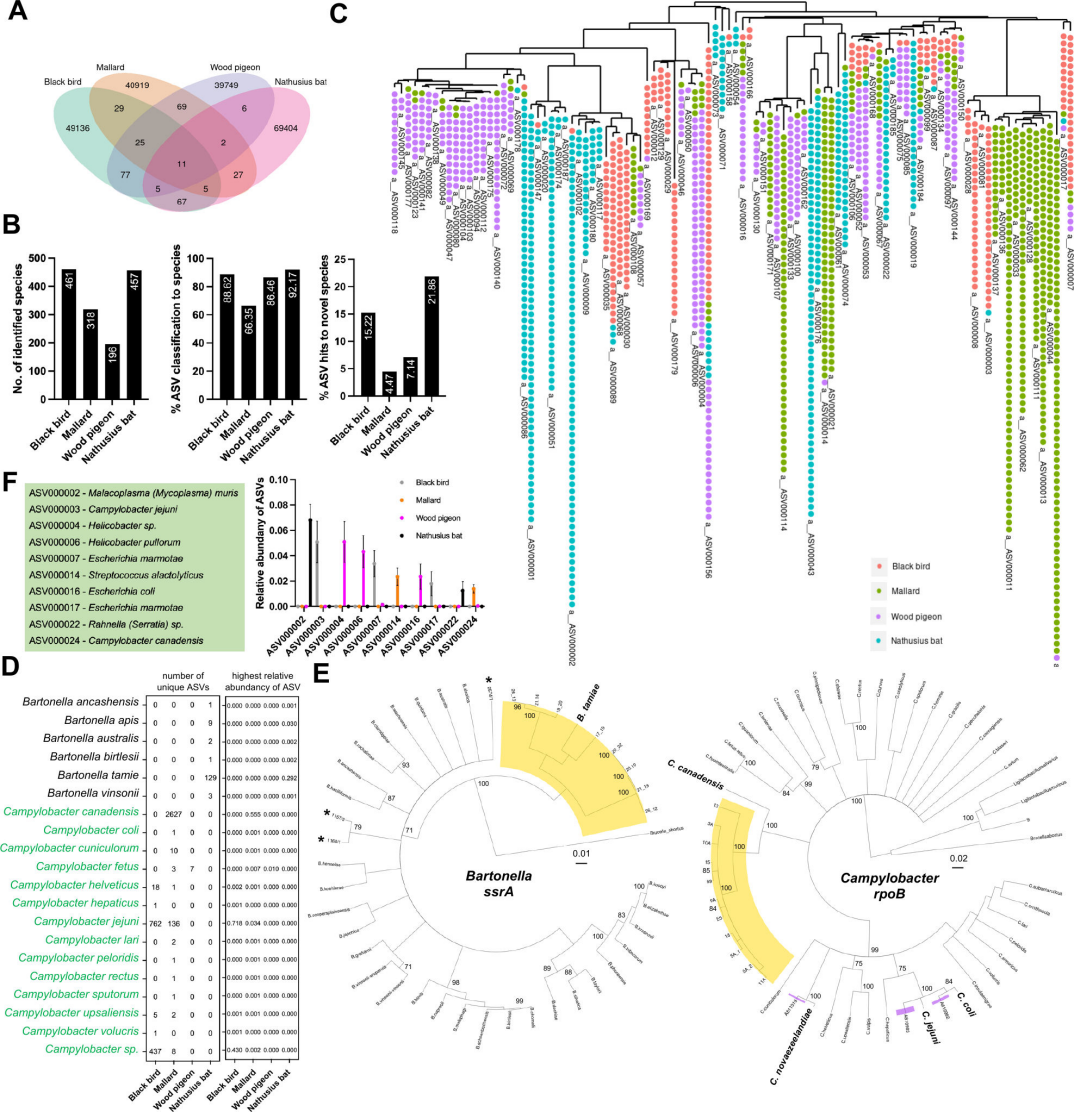
Dominance of host-specific human and animal pathogenic bacterial genera amplicon sequence variants. (A) The Venn diagram of unique and shared pASVs among the four host taxa, based on the entire data set without subsampling. The integer is the number of shared pASVs. (B) Description of the pASV taxonomic assignment to species level, based on the entire data set without subsampling. (C) The phylogenetic tree depicts the top 100 read count pASVs, based on the subsampled data set. Each colored point on the tree represents a single sample. (D) Example of host preference of pASVs, based on the subsampled data set. The numbers on the left refer to unique pASVs taxonomically assigned to the species level. The numbers on the right refer to the highest relative abundance value of a pASV (number of pASV-specific reads in a sample / number of all reads in a sample). (E) Phylogenetic trees to illustrate the similarities among *ssrA* sequences of different *Bartonella* species and *rpoB* sequences of different *Campylobacter* species with the sequences detected In this study. In the *ssrA* phylogeny, the clade highlighted in yellow indicates sequences exclusively found in Nathusius bat samples with similarity to *Bartonella tamiae*. The asterisk highlights *ssrA* sequences detected previously by us from samples of other bat species. In the *rpoB* phylogeny, the clade highlighted in yellow represents sequences solely identified in mallard samples with similarity to *Campylobacter canadensis*. Additionally, the clades highlighted in purple correspond to sequences found exclusively in blackbird samples, showcasing their proximity to *C. jejuni*, *Campylobacter coli*, and *Campylobacter novaezeelandiae*. Numbers on branches indicate bootstrap support values greater than 70 derived from 100 tree replicates. (F) Relative abundancy of pASVs in different host taxa (number of pASV-specific reads in a sample / all reads in a sample, mean and standard error of the mean, SEM), based on the subsampled data set. The figure displays 10 pASVs (refer to full data in File S5_52), which were detected in more than one host taxa also having statistically significant difference in relative abundancy to the other host taxa (multiple comparisons with analysis of variance, threshold 0.05).

A vast majority of pASVs were host taxa-specific when we analyzed the entire pASV data set without subsampling (Fig. 7A; File S3_41–41 and 4_40–43). Most of these pASVs could be assigned at species level (Fig. 7B). However, a considerable proportion of pASVs had hit to a novel species, that is, the top BLASTN hit was to a sequence designated in the SILVA database as sp. (Fig. 7B), for example, *Campylobacter* sp. RM16704. Phylogenetic analysis of 100 pASVs with the highest number of reads in the subsampled data set demonstrated that many pASVs indeed were host taxa-specific (Fig. 7C and D; File S3 and File S4_40–43). For example, ASV000179, identified as *Campylobacter jejuni*, was found in 19 samples of blackbird only (Fig. 7C and D; File S3_40). Likewise, ASV000061, identified as *Bartonella tamiae*, was found in 17 samples of Nathusius’ pipistrelle only (Fig. 7C and D, File S3_41–41 and 4_40–43). To substantiate the findings on host taxa specificity, we conducted a *Campylobacter-* and *Bartonella*-focused PCR and Sanger sequencing analysis of the entire fecal sample bank. We found that 32.7% (54 of 165) of blackbird, 89.6% (121 of 135) of mallard, and 2.5% (3 of 118) of pigeon samples were *Campylobacter* positive (File S5_44–47). Additionally, 9.9% (13 of 131) of Nathusius’ pipistrelle samples were *Bartonella* positive (File S5_44,48). The *Campylobacter rpoB* fragments were most similar with *C. jejuni* (File S5_49). The *Bartonella ssrA* fragments were most similar with *B. tamiae* (File S5_49). Sequence homology-based clustering of the Sanger-sequenced *rpoB* and *ssrA* fragments with *Campylobacter* and *Bartonella* type strain sequences (File S5_50), respectively, is shown in Fig. 7E. Finally, we conducted a statistical relative abundance analysis of those 324 pASVs, which were present in more than one host taxa (File S5_51). For example, ASV000007 (*Escherichia marmotae*) was detected exclusively in birds, but its relative abundance was highest in blackbird (0.034) (Fig. 7F; File S5_52). Overall, the data demonstrate that many pASVs were present in only one host taxa with high read numbers. If the pASV was present in more than one host taxa, it typically had a high relative abundancy in only one host taxa.

## DISCUSSION

In this study, we explored the fecal microbiota of three Eurasian migratory wild birds (mallard, blackbird, pigeon) and one Eurasian migratory bat (Nathusius’ pipistrelle). To the best of our knowledge, two previous microbiota studies have been published on cloacal swaps of mallard, but in a different continent (North America), utilizing the 16S rRNA V4 region sequencing with OTU bioinformatic approach (25, 26). The OTU approach clusters raw reads that differ by less than a fixed sequence dissimilarity threshold, most commonly by 3% (27). We employed the use of long-read PacBio sequencing of the nearly entire 16S rRNA gene (1,439 bp) (16). Furthermore, our downstream bioinformatic approach clustered raw read sequence variants differing up to two nucleotides into a single ASV. The ASV approach provides more taxonomic resolution as compared to the OTU approach (27). Overall, our data set contained 438,997 unique ASVs, most of which in low relative abundance and showing host taxa specificity. The ASVs allowed assignment of 3,277 bacterial species, as opposed to 2,205 species that would have been assigned based on the OTU approach. No bacterial taxon was present in all the host samples, not even at the bacterial phylum level. This means that each individual had quite a unique fecal microbiota.

Our FAPROTAX database (24) analysis indicated plant pathogenic potential of the fecal bacteria of Nathusius’ pipistrelle, but not so strongly of the other host taxa. The diet of Nathusius’ pipistrelle (28) is, to a large extent, restricted to insects associated with aquatic habitats, mainly Diptera. Therefore, the detected plant pathogenic potential most likely is due to plant-feeding insects. The finding appears interesting in light of the previous scarce literature on birds and bats as potential carriers of plant pathogens (29–31). Indeed, we detected ASVs, which were assigned to *Erwinia persicina* as well as *Pantoea agglomerans* and, less frequently, to *Erwinia rhapontici, Pseudomonas syringae,* and *Clavibacter michiganensis*. The last two are severe plant pathogens (32) and are, for example, included in the A2 list of quarantine bacteria of the European and Mediterranean Plant Protection Organization. Naturally, our study suffers from the same drawback as any DNA detection-based survey. The presence of DNA in a sample does not mean presence of viable let alone infective and disease-causing bacterium. Our data highlight the need for more elaborate molecular and culturing-based studies on Nathusius’ pipistrelle and plant pathogens in Europe.

Our FAPROTAX database (24) analysis indicated human pathogenic potential of the fecal bacteria of all host taxa. Therefore, out of all the bacterial genera, e.g., 19,153 genera based on the Genome Taxonomy Database estimate (https://gtdb.ecogenomic.org), we executed a sub-analysis with 66 pathogenic genera. Our list of 66 genera was based on the criteria that at least one species of each genera had to be pathogenic to human or animals. We also included several non-enteric genera to the list, e.g., vector transmitted *Bartonella* and *Borrelia*. Our previous studies have shown that fecal pellets can be used to monitor hemotrophic bacteria in bat populations (7), most likely due to ingestion of blood-sucking ectoparasites via grooming, and/or leakage of the gut epithelial barrier. The most important result of our analysis was the identification of pathogen bloomers in bat, blackbird and pigeon, but not in mallard. We define these pathogen bloomers as individuals where majority (>90%) of the PacBio reads were assigned to the list of 66 pathogenic genera. It was characteristic for these individuals that one single pASV had a high relative abundance value (>0.2), that is, the individual had a colonic pathogen dysbiosis. It is noteworthy that macroscopic disease indications of the pathogen bloomers were not reported to us during sampling. Therefore, it appears that the pathogen bloomers did not significantly suffer from the colonic pathogen dysbiosis. Given the Europe-wide migratory behavior of the sampled species, the pathogen bloomer-associated pathogens could quickly spread over long distances. Our data highlight the need for more elaborate follow-up studies on the pathogen bloomers as new risk factors of pathogen transmission across the European continent.

It is remarkable that individuals from higher latitudes had a higher pathogen load. Further research is needed to explore the ecological and physiological drivers of this phenomenon, e.g., via parallel profiling of the humoral and cell-mediated immunity and also viral load. However, the phenomenon could be related to the immunosuppressive stress experienced at the competitive high latitude breeding areas. It is noteworthy also that young individuals had a higher pathogen load. This could be related to their premature immune system, in particular the humoral component, but other drivers such as differential diet might contribute. Recent work in UK on mute swan (*Cygnus olor*) demonstrated that some bacterial taxa indeed were differentially detected in different age groups (33). It appears that our research field should more widely consider the age of the sampled individuals and, for example, study how shifts in breeding-success and age-specific mortality might impact the risks of bacterial pathogen spillover.

Our sub-analysis of the 66 pathogenic genera led into important species-level taxonomic assignments. It is important to analyze this taxonomic level, because some analyzed bacterial genera also have species with a weak to negligible pathogenicity, e.g., *Actinomyces*. In blackbird, three well-known enteric pathogens were frequently detected, that is, *Campylobacter jejuni* (56.8, 762, 0.7180, 0.5265, all countries), *Escherichia coli* (54.3, 725, 0.24, 0.1159, all countries), and *Clostridium perfringens* (12.3, 119, 0.0660, 0.0272, all countries except Latvia). The numbers in brackets refer to the percentage of positive samples, number of unique ASVs, highest relative abundancy value of an ASV, and mean of the top 10 highest ASV relative abundancy values, respectively (subsampled data set). Interestingly, blackbird was rich of unique ASVs specific for the emerging enteric pathogen *Escherichia marmotae* in all countries (44.4, 1284, 0.6760, 0.3310). The type strain of *Escherichia marmotae* has been isolated from the feces of a wild marmot (*Marmota himalayana*) in China (34). Some other isolates with troubling antibiotic resistance properties have been reported, e.g., from cattle in Spain (35) and from common starling (*Sturnus vulgaris*) in Tunisia (36). Recently, the first invasive human cases of *Escherichia marmotae* were described in Norway (37). In mallard, three well-known enteric pathogens were frequently detected both in Finland and France, that is, *Escherichia coli* (30.5, 234, 0.059, 0.01990), *Campylobacter jejuni* (42.6, 136, 0.034, 0.02880), and *Clostridium perfringens* (16.7, 137, 0.0380, 0.0174). Interestingly, mallard had several ASVs specific for *Streptococcus suis* (13.0, 21, 0.013, 0.0032) both in Finland and France. This frequent colonizer of the upper respiratory tract of pigs causes global economic problems for agriculture (38). Moreover, it is a zoonotic agent causing severe human infections with large outbreaks reported in China (38). Recently, the carriage of *Streptococcus suis* in chicken flocks in Vietnam was reported (39), but the potential of wild birds as the reservoir has remained unknown. Pigeon was extremely rich of unique ASVs specific for *Escherichia coli* (69.3, 1747, 0.474, 0.3284), but other well-known or emerging pathogens were scarce. In bat, two well-known enteric pathogens were frequently detected, that is, *Vibrio cholerae* (22.7, 377, 0.3250, 0.0980, in Finland and Latvia) and *Escherichia coli* (23.9, 255, 0.463, 0.1425, all countries). Interestingly, only two samples in Latvia were positive for *Vibrio cholerae*, in contrast to 18 Finnish samples, although the analyzed sample numbers were almost equal. An opposite pattern was seen with *Bartonella tamiae* (28.4, 129, 0.2960, 0.05980), an emerging human pathogen (40), as four Finnish and 21 Latvian samples were positive for *Bartonella tamiae*. The pathogen-focused sub-analysis across all the host taxa also led to single ASV and low relative abundance taxonomic assignments to well-known enteric pathogens, e.g., *Shigella flexneri*, *Shigella dysenteriae*, and *Yersinia pseudotuberculosis*, but also to well-known non-enteric pathogens, e.g., *Haemophilus influenzae*, *Streptococcus pneumoniae*, *Neisseria meningitidis*, *Klebsiella pneumoniae*, *Staphylococcus aureus*, and *Acinetobacter baumannii*. However, these findings, in particular, the non-enteric hits, require more validation, e.g., with targeted PCR- and culturing-based studies. It is noteworthy also that we got multiple top hits to sp. status bacteria, that is, novel species. One example in blackbird was *Campylobacter* sp. RM16704 frequently detected in all countries (40.7, 429, 0.4300, 0.2942). The type strain has been isolated from fresh water samples in the USA, and based on its genomic properties, it categorizes as a novel taxon within *Campylobacter* (41). Overall, our species level sub-analysis indicates that the fecal samples contained bacteria with pathogenic potential.

Identifying the wildlife reservoirs of bacterial pathogens, spatially and temporally, is important for assessing the threats to human and the rest of the biosphere. Our data imply that the studied highly mobile and common migratory vertebrates have a potential role in the transmission of a number of well-known or emerging bacterial pathogens across the European continent. These data may contribute to better understanding of seasonal disease hotspots. Our data set also revealed a vast amount of new bacterial taxonomic information. We expect that these data improve diagnostic capabilities and, in the future, allow for a more comprehensive understanding of pathogen carriage in wildlife, and detection of clinical cases. Lastly, we anticipate that the increased knowledge of pathogen prevalence in Europe will contribute to more efficient modeling approaches, e.g., to understand the connections between the changing climatic variables and pathogen spillover risks.

## Data Availability

The data sets generated during the current study are available in the NCBI repository. The raw PacBio sequencing data are available at BioProject accession no. PRJNA977964. The edited MLSA loci sequences are available at OQ873545-OQ873558 and OQ877073-OQ877081.

## SUPPLEMENTAL MATERIAL

Data S1 spectrum.01948-24-s0001.xlsx 1 to 35.

Data S2 spectrum.01948-24-s0002.xlsx 36 to 39.

Data S3 spectrum.01948-24-s0003.xlsx 40 and 41.

Data S4 spectrum.01948-24-s0004.xlsx 42 and 43.

Data S5 spectrum.01948-24-s0005.xlsx 44 to 52.

Supplemental figures spectrum.01948-24-s0006.pdf Fig. S1 to S5.

Supplemental material spectrum.01948-24-s0007.pdf Description of the PacBio sequence analysis pipeline.

ASM does not own the copyrights to Supplemental Material that may be linked to, or accessed through, an article. The authors have granted ASM a non-exclusive, world-wide license to publish the Supplemental Material files. Please contact the corresponding author directly for reuse.
