# Supplementary material for: Pathogenic bacterial taxa constitute a substantial portion of fecal microbiota in common migratory bats and birds in Europe
Source: Microbiol Spectr. 2025 Feb 4;13(3):e01948-24. doi: 10.1128/spectrum.01948-24 (PMC11878047; doi:10.1128/spectrum.01948-24)
Supplement: Supplemental figures — Fig. S1 to S5. [file spectrum.01948-24-s0006.pdf]

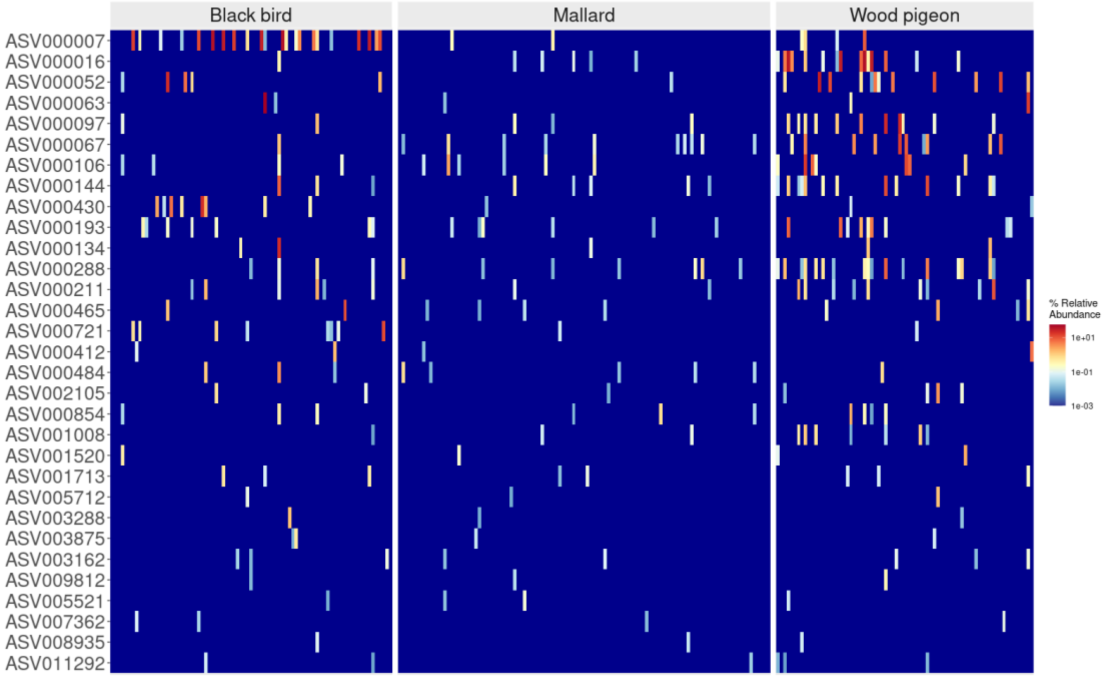

Figure S1. Poosakkannu et al.

Supplementary Figure 1. Amplicon sequence variants found in all the bird host taxa. Heat map of the % relative abundancies (number of amplicon sequence variant [ASV] specific reads in a sample / all reads in a sample *100) of the 31 ASVs detected in at least one individual of all the bird taxa.

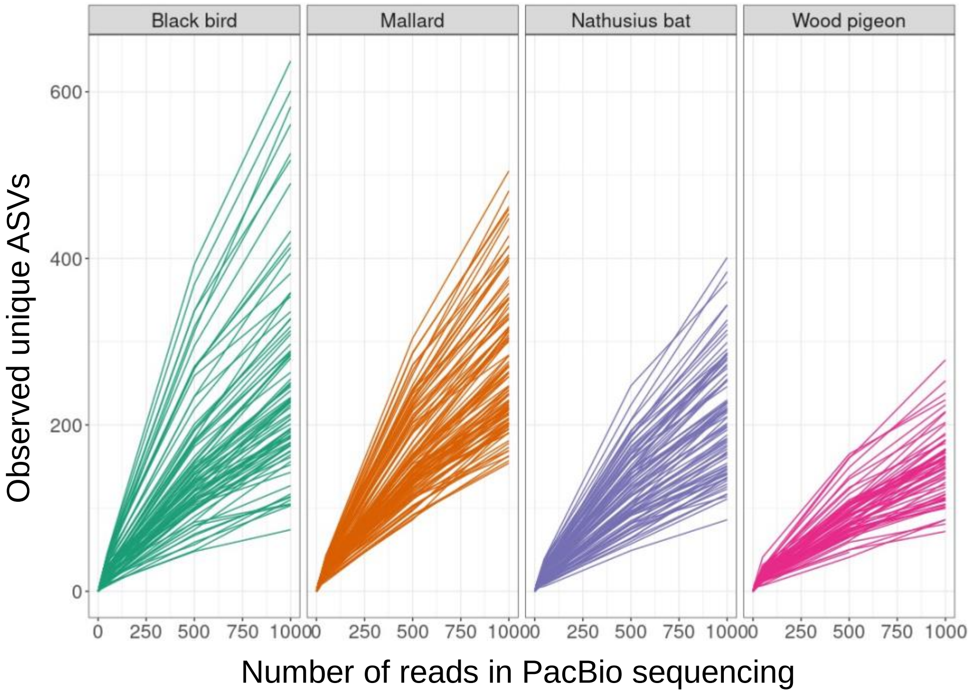

Figure S2. Poosakkannu et al.

Supplementary Figure 2. The rarefaction analysis. The X-axis refers to the number of total sequences observed in a particular sample. The Y-axis refers to the number of unique amplicon sequence variants (ASVs) detected in a particular sample. According to the rarefaction analysis and Good's coverage analysis (Suppl. file 1_9), using subsampling for 1,000 sequences per sample was deemed sufficient to adequately represent most of the bacterial diversity. The subsampling removed 10 samples because they contained fewer reads than 1,000 sequences, i.e., the final dataset is composed of 339 samples.

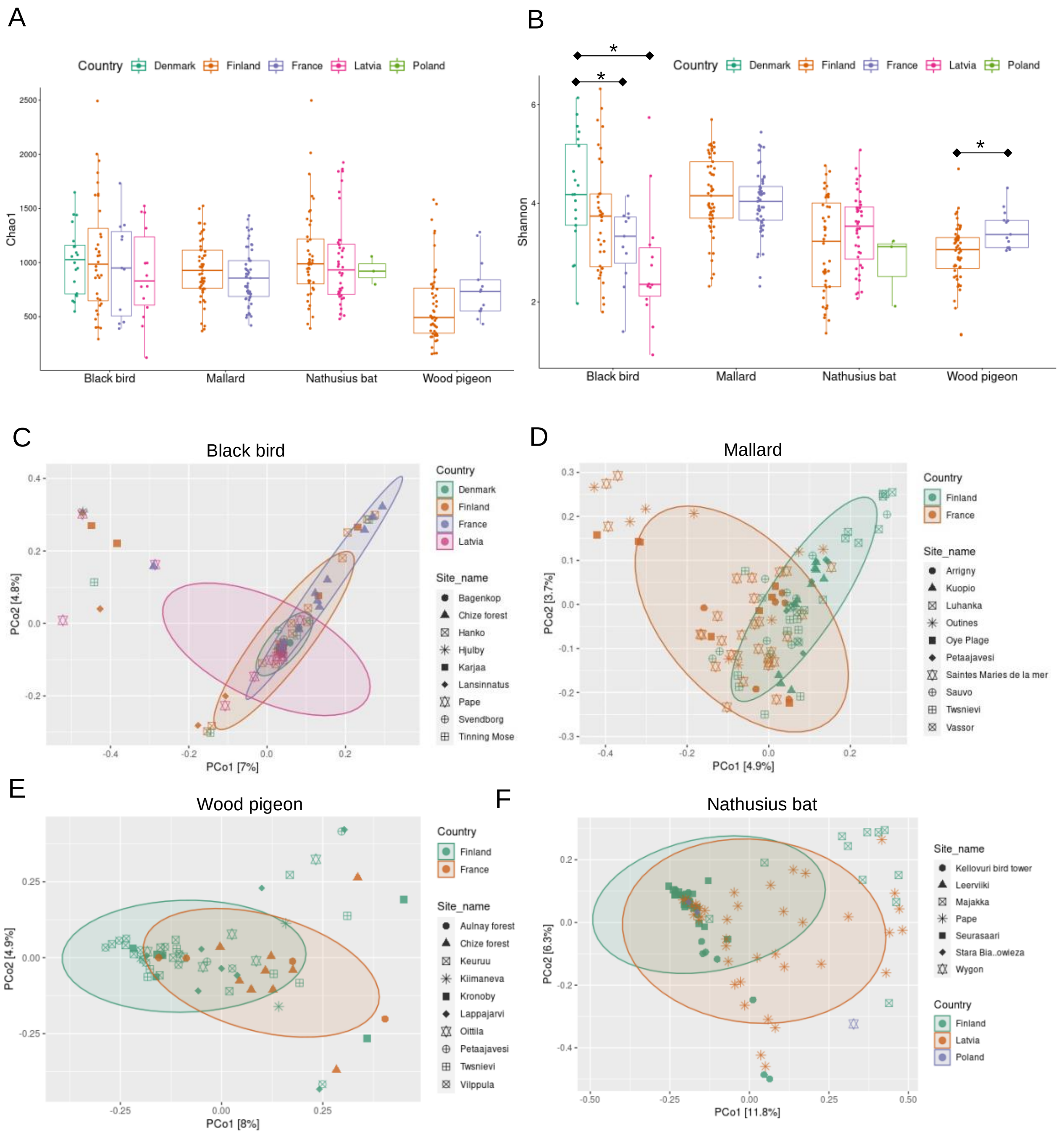

Figure S3. Poosakkannu et al.

Supplementary Figure 3. The effect of country on fecal microbiota composition. The box plots of the Chao1 **(A)** and **(B)** Shannon diversity indices in country groups of different host taxa based on the subsampled ASV data set are displayed. Each point represents a single sample. The significance estimates were calculated for the country by Wilcox rank sum test. Asterisks designate significance level (***≤ 0.001, **≤ 0.01, *≤ 0.05). The PCoA ordination plot for the country groups of the black bird **(C)**, mallard **(D)**, pigeon **(E)**, and Nathusius' pipistrelle **(F)**. Each point represents a single sample. The ellipses are drawn based on the 95% confidence intervals.

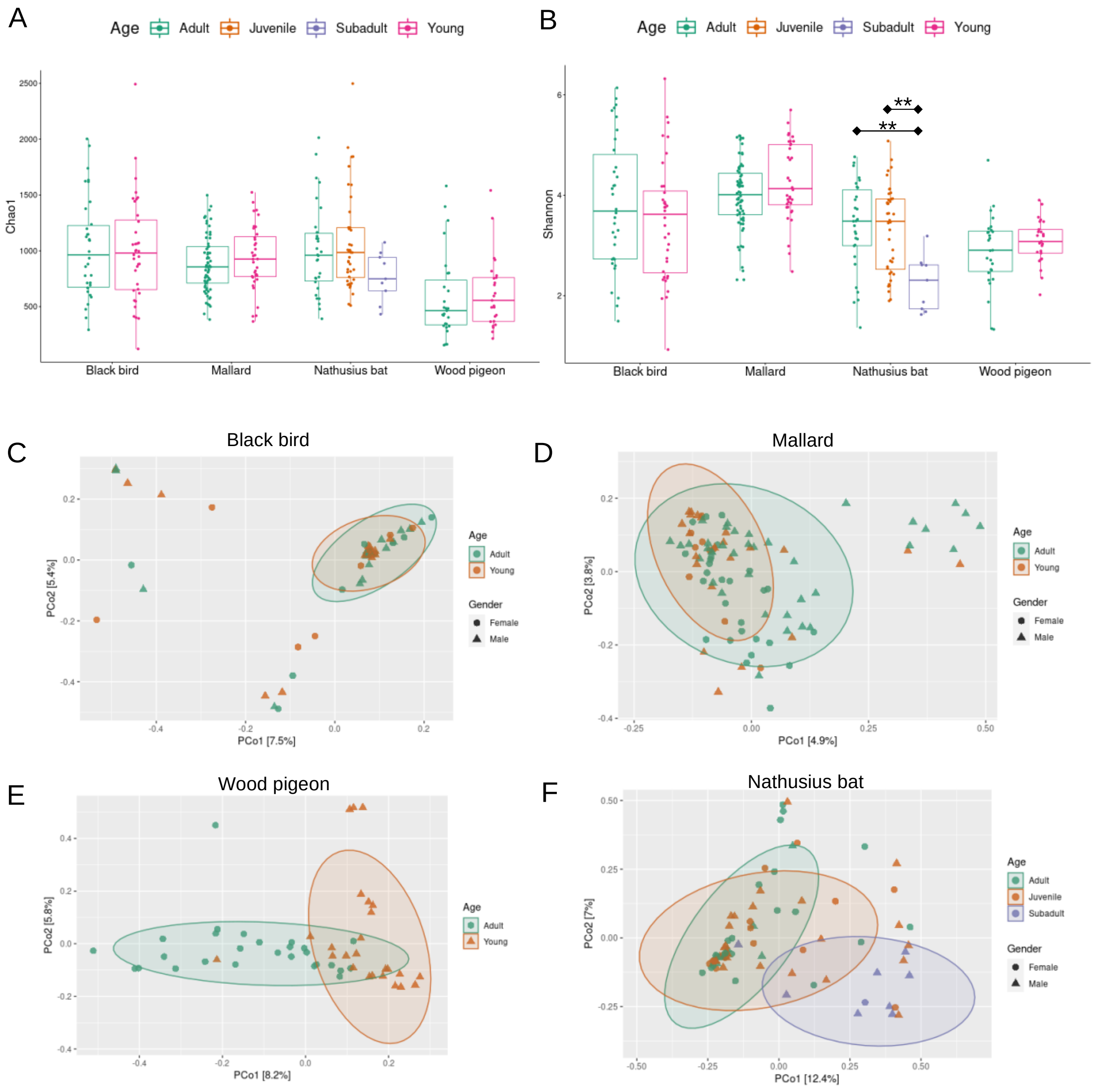

Figure S4. Poosakkannu et al.

Supplementary Figure 4. The effect of age on fecal microbiota composition. The box plots of the Chao1 **(A)** and **(B)** Shannon diversity indices in age groups of different host taxa based on the subsampled ASV data set are displayed. Each point represents a single sample. The significance estimates were calculated for the age by Wilcox rank sum test. Asterisks designate significance level (***≤ 0.001, **≤ 0.01, *≤ 0.05). The PCoA ordination plot for different age groups of the black bird **(C)**, mallard **(D)**, pigeon **(E)**, and Nathusius' pipistrelle **(F)**. Each point represents a single sample. The ellipses are drawn based on the 95% confidence intervals.

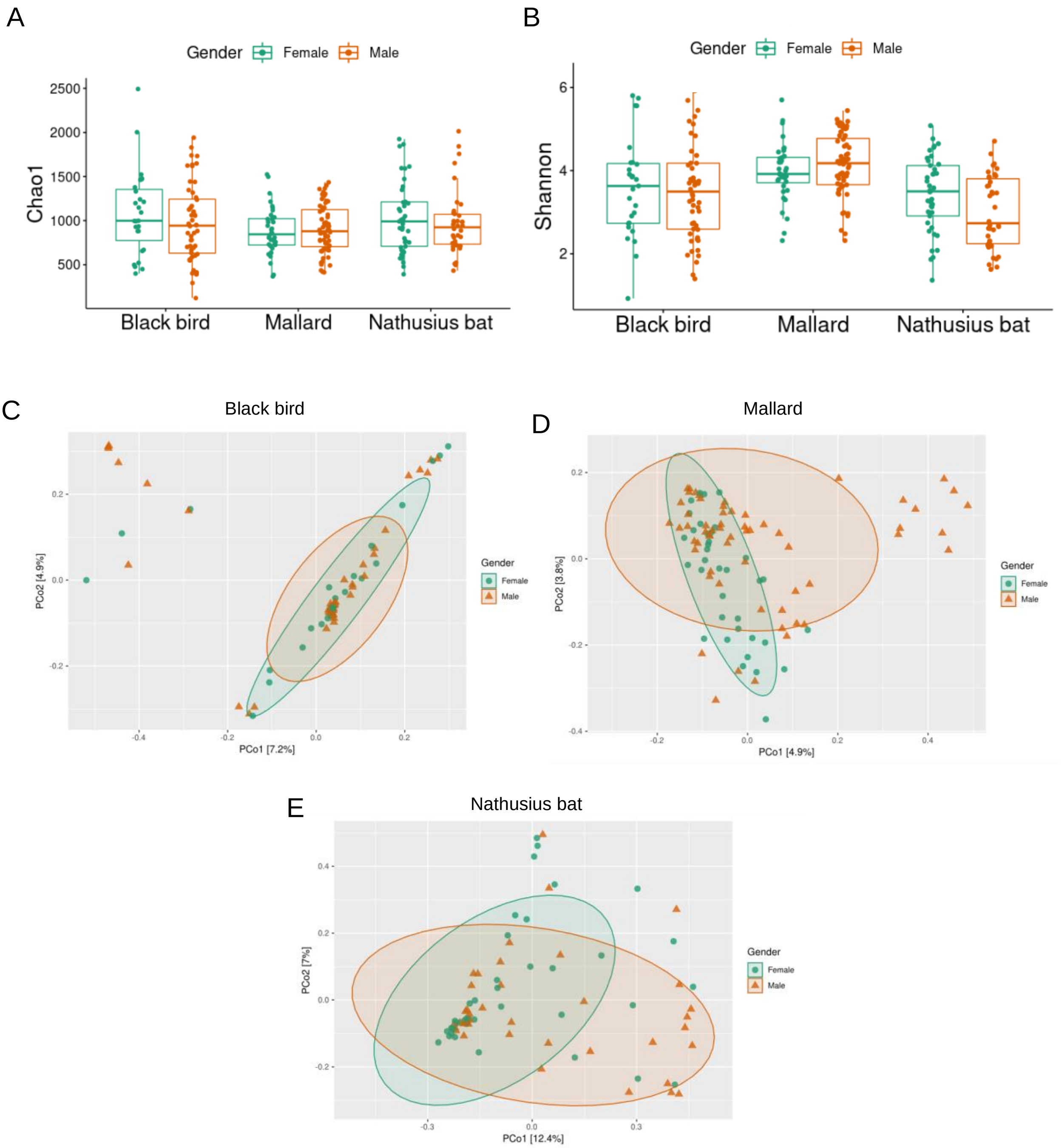

Figure S5. Poosakkannu et al.

Supplementary Figure 5. The effect of gender on fecal microbiota composition. The box plots of the Chao1 **(A)** and **(B)** Shannon diversity indices in groups of different host taxa based on the subsampled ASV data set are displayed. Each point represents a single sample. The significance estimates were calculated for the gender by Wilcox rank sum test. Asterisks designate significance level (***≤ 0.001, **≤ 0.01, *≤ 0.05). The PCoA ordination plot for different gender groups of the black bird **(C)**, mallard **(D)** and Nathusius' pipistrelle **(E)**. Of note, we did not have gender information of pigeon. Each point represents a single sample. The ellipses are drawn based on the 95% confidence intervals.
