# Supplementary material for: Pathogenic bacterial taxa constitute a substantial portion of fecal microbiota in common migratory bats and birds in Europe
Source: Microbiol Spectr. 2025 Feb 4;13(3):e01948-24. doi: 10.1128/spectrum.01948-24 (PMC11878047; doi:10.1128/spectrum.01948-24)
Supplement: Supplemental material — Description of the PacBio sequence analysis pipeline. [file spectrum.01948-24-s0007.pdf]

Description of the PacBio sequence analysis pipeline

Mothur Pipeline for Processing PacBio Sequences
This pipeline processes raw PacBio FASTQ files, generates ASV abundance tables, and retrieves representative ASV sequences.

Step 1: Extract Sequence Information
Extract metadata from PacBio-generated FASTQ files for 349 samples and two blank controls. Below is an example command with placeholder sample names. To run the command, replace the sample names with the correct file paths for your specific dataset.

```bash
fastq.info(fastq=sample1.fastq-sample2.fastq-...-sample349.fastq-blank1.fastq-
blank2.fastq, pacbio=T)
```

Step 2: Generate a Group File
Create a group file associating each sample's sequences. Below is an example command with placeholder sample names. To run the command, replace the sample names with the correct file paths for your specific dataset.

```bash
make.group(fasta=sample1.fasta-sample2.fasta-...-sample349.fasta-blank1.fasta-
blank2.fasta, groups=sample1-sample2-...-sample349-blank1-blank2)
```

Step 3: Merge FASTA Files
Combine all FASTA files into a single file. Below is an example command with placeholder sample names. To run the command, replace the sample names with the correct file paths for your specific dataset.

```bash
merge.files(input=sample1.fasta-sample2.fasta-...-sample349.fasta-blank1.fasta-
blank2.fasta, output=merged_samples.fasta)
```

Step 4: Summarize and Screen Sequences
Summarize and remove sequences with ambiguous bases and long homopolymer regions.

```bash
summary.seqs(fasta=merged_samples.fasta)
screen.seqs(fasta=merged_samples.fasta, maxambig=0, maxhomop=8)
```

Step 5: Generate and Align Unique Sequences
Identify unique sequences and align them to the SILVA reference database.

```bash
unique.seqs(fasta=merged_samples.good.fasta)
align.seqs(fasta=merged_samples.good.unique.fasta, reference=silva.nr_v138_2.align)
```

Step 6: Screen, Filter, and Pre-cluster
Screen based on alignment positions, filter gaps, and pre-cluster sequences to reduce noise.

```bash
screen.seqs(fasta=merged_samples.good.unique.align, start=1044, end=43116)
filter.seqs(fasta=merged_samples.good.unique.good.align, vertical=T, trump=.)
pre.cluster(fasta=merged_samples.good.unique.good.filter.fasta, diffs=2)
```

Step 7: Detect and Remove Chimeras
Detect chimeric sequences using **VSEARCH** and remove them.

```bash
chimera.vsearch(fasta=current, dereplicate=T)
remove.seqs(fasta=current, accnos=current)
```

Step 8: Create Shared and Representative Files
Generate the ASV abundance table and retrieve representative ASV sequences.

```bash
make.shared(count=current)
get.oturep(fasta=current, list=current, count=current, rename=T)
```

Taxonomic Assignment using SILVA Database

Step 1: Download and Prepare the SILVA Database
Create a BLAST database from the SILVA reference file.

```bash
makeblastdb -in SILVA_138_SSURef_Nr99_tax_silva_trunc.fasta -dbtype nucl -out
SILVA_138_NR99
```

Step 2: Assign Taxonomy
Perform BLAST searches for taxonomic assignment.

```bash
blastn -query representative_ASVs.fasta -db SILVA_138_NR99 \
-outfmt "7 qseqid stitle" -max_target_seqs 1 -num_threads 10 \
-out taxonomy_assignment.out
```

Step 3: Format Taxonomic Output
Clean and format the BLAST output for downstream analysis.

```bash
sed '/^#/ d' taxonomy_assignment.out > formatted_taxonomy.txt
sed '1 i\#ASVID\ttaxonomy' -i formatted_taxonomy.txt
sed -r 's/(ASV_[0-9]+)(\t[[:alnum:].]+[ ]+)/\1\tD_0__/' -i formatted_taxonomy.txt
sed -E 's/;/;D_1__/1;s/;/;D_2__/2;s/;/;D_3__/3;s/;/;D_4__/4;s/;/;D_5__/5;s/;/;D_6__/6' -i
formatted_taxonomy.txt
```

Output Format Example:
**ASVID**	**Taxonomy**
ASV_1	D_0__Bacteria;D_1__Proteobacteria;D_2__Gammaproteobacteria;D_3__Enterobacteriales;D_4__Enterobacteriaceae
ASV_2	D_0__Archaea;D_1__Euryarchaeota;D_2__Methanobacteria;D_3__Methanobacteriales

---

QIIME 2 Pipeline for Phylogenetic Tree Construction

Step 1: Import Aligned Sequences
Import representative ASVs into a QIIME 2 artifact.

```bash
qiime tools import \
  --input-path representative_ASVs_aligned.fasta \
  --output-path representative_ASVs.qza \
  --type 'FeatureData[AlignedSequence]'
```

Step 2: Build an Unrooted Phylogenetic Tree
Generate a phylogenetic tree using FastTree.

```bash
qiime phylogeny fasttree \
  --i-alignment representative_ASVs.qza \
  --o-tree unrooted_tree.qza
```

Step 3: Root the Phylogenetic Tree
Root the tree at its midpoint.

```bash
qiime phylogeny midpoint-root \
  --i-tree unrooted_tree.qza \
  --o-rooted-tree rooted_tree.qza
```

Step 4: Export the Phylogenetic Tree
Export the rooted tree in Newick format.

```bash
qiime tools export \
  --input-path rooted_tree.qza \
  --output-path rooted_tree
```

Final Output Files
1. **ASV Abundance Table** (`asv_abundance_table.shared`)
2. **Representative ASV Sequences** (`representative_ASVs.fasta`)
3. **Taxonomy Assignments** (`formatted_taxonomy.txt`)
4. **Rooted Phylogenetic Tree** (`rooted_tree/tree.nwk`)
